# Quantifying Replication Slippage Error in *Cryptosporidium* Metabarcoding Studies

**DOI:** 10.1093/infdis/jiae065

**Published:** 2024-02-08

**Authors:** Matthew A Knox, Patrick J Biggs, Juan Carlos Garcia-R, David T S Hayman

**Affiliations:** School of Veterinary Science; School of Veterinary Science; School of Natural Sciences, Massey University, Palmerston North, Manawatu-Wanganui, New Zealand; School of Veterinary Science; School of Veterinary Science

**Keywords:** *Cryptosporidium hominis*, *Cryptosporidium parvum*, dada2, PCR slippage, Short Tandem Repeat

## Abstract

Genetic variation in *Cryptosporidium*, a common protozoan gut parasite in humans, is often based on marker genes containing trinucleotide repeats, which differentiate subtypes and track outbreaks. However, repeat regions have high replication slippage rates, making it difficult to discern biological diversity from error. Here, we synthesized *Cryptosporidium* DNA in clonal plasmid vectors, amplified them in different mock community ratios, and sequenced them using next-generation sequencing to determine the rate of replication slippage with dada2. Our results indicate that slippage rates increase with the length of the repeat region and can contribute to error rates of up to 20%.

## Teaser

DNA repeat regions differentiate subtypes and track *Cryptosporidium* outbreaks but have high replication slippage rates. Using synthesized DNA, we demonstrate that slippage rates increase with the length of repeat region and can contribute to error rates of up to 20%.

Replication slippage, also known as slipped strand mispairing, is a natural mutation process that occurs during DNA replication. It involves the misalignment of DNA strands, leading to the addition or deletion of a repeated sequence of nucleotides in a specific region of the DNA [1]. This phenomenon is particularly common in repetitive DNA sequences, such as microsatellites or short tandem repeats (STRs), which are stretches of DNA where a short sequence of nucleotides is repeated multiple times in a row. This phenomenon has implications for genetic diversity, evolution, and the development of certain genetic diseases [2] and can be measured by amplification and sequencing of the repeat region.

The most commonly used marker for characterizing diversity in intestinal protozoan parasite *Cryptosporidium* is the 60 kDa glycoprotein (gp60) gene, which contains nucleotide sequence differences, trinucleotide repeats, and other repeat regions [3]. The trinucleotide repeats encode serines that are used to classify subtypes within subtype families. Sanger sequencing is sufficient for identifying the dominant subtype family in a sample, but recent studies using next-generation sequencing (NGS) have revealed hitherto unrecognized diversity in *Cryptosporidium* infections and provided valuable insights into the epidemiology and biology of these parasites [4, 5]. Outbreaks seldomly appear to be caused by a single *Cryptosporidium* subtype, and host coinfection with multiple subtype families or even by different species appears to be possible [6]. However, amplification of short repeats in gp60 amplicons is susceptible to DNA polymerase slippage during polymerase chain reaction (PCR) elongation steps [7]. This makes it difficult to distinguish true diversity (and possible rare or emerging *Cryptosporidium* subtypes with epidemiological relevance) from artifacts resulting from sequencing errors including replication slippage during PCR amplification or contamination.

Previous studies have evaluated the suitability of gp60 metabarcoding [6] using samples naturally infected with *Cryptosporidium*. However, the lack of appropriate pure culture controls, time-consuming procedures (such as amplicon cloning) on large numbers of isolates, and inaccuracies in laboratory and sequencing processes have hampered efforts to quantify error rates and remove experimental noise from meaningful results. Therefore, directly assessing error rates associated with *Cryptosporidium* gp60 gene metabarcoding has not previously been undertaken. To address this issue, we designed and synthesized *Cryptosporidium* gp60 gene fragments that match subtypes from *Cryptosporidium hominis* (IbA10G2) and *Cryptosporidium parvum* (IIdA19G1). We then conducted mock community pre- and post-PCR experiments to assess the accuracy of metabarcoding for assessing *Cryptosporidium* diversity.

## METHODS

Synthesized material was created by Genescript, inserted into cloned vectors (pUC57), and extracted to provide a pure culture of each subtype with a total length of 3212 bp for *C hominis* and 3171 bp for *C parvum*, due to the different sizes of each species-specific insert. Both insert sequences contained modifications to the original template sequence to distinguish them from potential contaminating *Cryptosporidium* DNA. The *C hominis* IbA10G2 insert was 502 bp and contained a repeat region with 13 serines, whereas the one for *C parvum* IIdA19G1 was 461 bp and had 20 serine repeats. The synthetic sequences were used to create mock communities for testing sequencing error rates as well as variation and biases in PCR.

Extracted plasmid samples (see Supplementary Information) were quantified using Qubit 1X dsDNA HS Assay Kit (Thermo Fisher, Waltham, Massachusetts) and diluted to normalized concentrations. Copy number calculations were performed to account for the slightly larger *C hominis* amplicon and resulted in a known number of gp60 copies added to each PCR reaction (2 × 10^6^). Nested PCR amplification of extracted products followed previous protocols [5] and used Platinum Taq DNA polymerase (Invitrogen). The internal primers were modified to contain MiSeq adapter sequences on the 5′ end according to standard protocols [8]. PCR clean-ups used Ampure beads and ethanol washes. Cleaned samples were quantified using Qubit and normalized to approximately 5 ng/µL. The slight size difference of amplicons (*C hominis* was 41 bp longer) was accounted for by calculating and equalizing the amplicon copy number such that a known number of gp60 copies was present per microliter of *C hominis* and *C parvum* samples.

Amplified material from pure *C hominis* and *C parvum* subtypes was combined after PCR in triplicate samples and in different percentage combinations including pure *C hominis* (100:0) and *C parvum* (0:100) as well as 99:1, 75:25, 50:50, 25:75, and 1:99 copy number combinations (post-PCR samples, Table 1). In addition, to test for PCR biases between taxa in mixed template reactions, triplicate reactions of samples in the 99:1, 75:25, 50:50, 25:75, and 1:99 copy number combinations were added to PCR reactions (pre-PCR samples, Table 1).

**Table 1.** Polymerase Chain Reaction Experimental Design and Read Count Results (Average of 3 Replicates) From Map to Reference Analyses of Forward Reads

	PCR Mix (*C hominis: C parvum*)	Sample Number	Template Copy Number Added to PCR		Template Percentage		Read Count		Percent Read Count		Difference From Expected Percent Read Count	
			*C hominis*	*C parvum*	*C hominis*	*C parvum*	*C hominis*	*C parvum*	*C hominis*	*C parvum*	*C hominis*	*C parvum*
Post-PCR	100:000	1–3	2 000 000	0	100%	0%	8640.0	5.0	99.9%	0.1%	−0.1%	0.1%
	000:100	4–6	0	2 000 000	0%	100%	8.7	8847.7	0.1%	99.9%	0.1%	−0.1%
	050:050	7–9	NA^a^	NA^a^	50%	50%	4494.3	4893.7	47.7%	52.3%	−2.3%	2.3%
	075:025	10–12	NA^a^	NA^a^	75%	25%	6553.0	2611.3	71.5%	28.5%	−3.5%	3.5%
	025:075	13–15	NA^a^	NA^a^	25%	75%	2243.3	6335.7	26.1%	73.9%	1.1%	−1.1%
	099:001	16–18	NA^a^	NA^a^	99%	1%	7990.7	125.3	98.5%	1.5%	−0.5%	0.5%
	001:099	19–21	NA^a^	NA^a^	1%	99%	105.3	8621.7	1.2%	98.8%	0.2%	−0.2%
Pre-PCR	050:050	22–24	1 000 000	1 000 000	50%	50%	4585.3	4397.3	51.0%	49.0%	1.0%	−1.0%
	075:025	25–27	1 500 000	500 000	75%	25%	3338.7	2586.0	56.4%^b^	43.6%^b^	−18.6%	18.6%
	025:075	28–30	500 000	1 500 000	25%	75%	3788.7	4151.0	47.7%^b^	52.3%^b^	22.7%	−22.7%
	099:001	31–33	1 980 000	20 000	99%	1%	5612.7	2331.7	70.4%^b^	29.6%^b^	−28.6%	28.6%
	001:099	34–36	20 000	1 980 000	1%	99%	2738.7	5143.0	34.8%^b^	65.2%^b^	33.8%	−33.8%
Controls	N/A	37–38	0	0	0%	0%	0.0	0.0	0.0%	0.0%	0.0%	0.0%

Abbreviations: *C hominis*, *Cryptosporidium hominis*; *C parvum*, *Cryptosporidium parvum*; N/A, not applicable; PCR, polymerase chain reaction.

^a^Samples were derived from amplicons generated from samples 1–6 and combined before library preparation.

^b^Indicates *P* < .001 from χ^2^ goodness-of-fit test using expected and observed percent read counts.

Sequencing was carried out on an Illumina MiSeq using 500-cycle V2 chemistry, according to the manufacturer's recommendations, producing 2 × 250 base paired-end reads. An Illumina PhiX control library was loaded onto the run at 20% volume, to even out the base composition and prevent biases. All samples have been deposited to the National Center for Biotechnology Information Sequence Read Archive under Bio-project PRJNA1013320 with accession numbers SAMN37295715–SAMN37295752.

Reads were trimmed using DynamicTrim application (http://solexaqa.sourceforge.net/) with the quality cutoff set at 0.01. To examine variation in the repeat region only, forward reads were trimmed to minimum length of 200 bp and mapped to references based on synthetically designed *C hominis* and *C parvum* sequences using Geneious (v.10.2.6) with low sensitivity. The observed proportion of reads belonging each species were compared to the expected ratios and tested using goodness-of-fit test with 99% confidence level. The contig(s) were then further trimmed to only include the serine repeat regions, then the length distribution of all reads was recorded.

To compare with previous studies, we also analyzed the data using amplicon approaches on the paired reads. The Illumina sequence reads for the 36 samples involved in this study were analyzed using R (v.4.2.2) and dada2 (v.1.26.0) [9], tidyverse (v.1.3.2), ggplot2 (v.3.4.2), phyloseq (v.1.42.0), and ShortRead (v.1.56.1) packages. A default dada2 pipeline approach was taken to filter and trim the forward and reverse sequence reads, de-replicate them, calculate and plot error rates, merge paired reads and construct a sequence table, and remove chimeras. We initially ran dada2 with default settings and then with the BANDSIZE value set to “2,” whereas the default value is “16.” The change in the BANDSIZE was chosen to be the largest value less than the triplet size, so that individual triplets in the repeat region resulted in discrete amplicons being kept within the analysis. Code is available on Github (https://github.com/pjbiggs/CryptosporidiumSlippage). In addition, the analysis was performed with the 20 most abundant amplicons, which accounted for 99.7% of the sequence reads in the study.

## RESULTS

**Figure 1.**
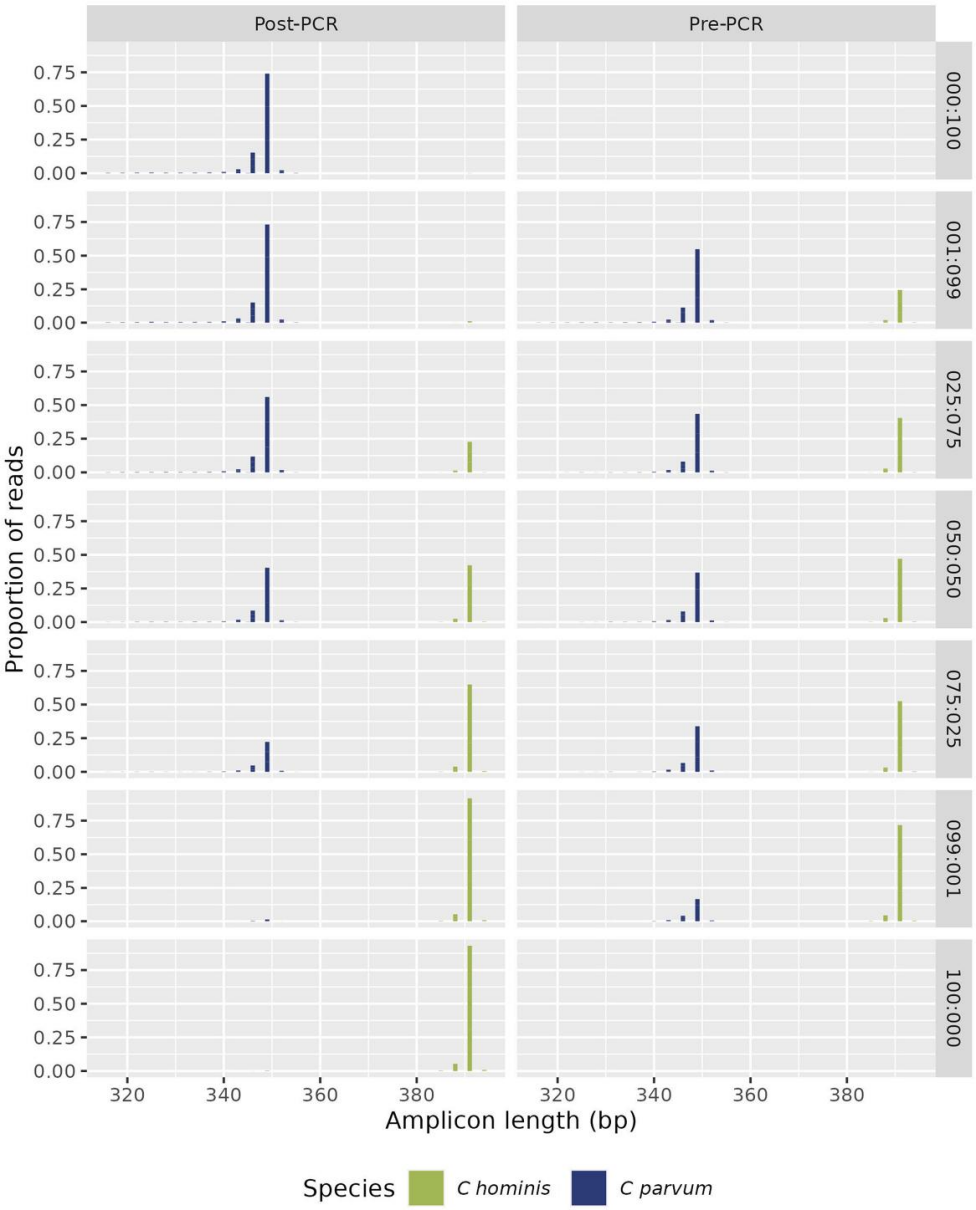
Amplicon length proportions for combinations of species (on right of charts where ratios refer to different combinations of *Cryptosporidium hominis* and *Cryptosporidium parvum* template). Post– and pre–polymerase chain reaction (PCR) treatments refer to when the *C hominis* and *C parvum* template combinations occurred. The expected trimmed amplicon length was 391 and 349 bp for *C hominis* and *C parvum*, respectively.

Average read counts for each sample type are recorded in Table 1. Cross-contamination rates between species were <0.01%. Post-PCR combinations of *C hominis* IbA10G2 and *C parvum* IIdA19G1 templates matched the expected proportions after classification with map to reference analyses using Geneious. In contrast, the percent read counts for the mixed community amplifications (pre-PCR) did not match the template percentages in samples that were unbalanced—that is, the 75:25 and 99:1 combinations. In lower template concentrations, both *C hominis* and *C parvum* templates were overrepresented by between 18.6% and 33.8% (*P* values <.001 from χ^2^ test; Figure 1, Table 1).

Serine repeat regions varied in length in both templates and consistently across all sample types in the Geneious analysis. In the *C hominis* template, 95.5% of reads were of the expected size, with 3.8% being 1 serine (3 bases) shorter and 0.7% being 1 serine longer. The same pattern was seen in *C parvum*, only more pronounced, with 80% of reads of the expected size, 16.6% being 1 serine (3 bases) shorter, 1.8% being 2 serines (6 bases) shorter, and 1.6% being 1 serine longer (Supplementary Figure 1). The dada2 pipeline approach generated the same results as above, with higher rates of replication slippage in the *C parvum* amplicon and overrepresentation of lower concentration templates in the pre-PCR samples (Figure 1).

## DISCUSSION

Our results demonstrate that contamination from different subtype families was absent in the metabarcoding and that PCR and NGS recover the sequences inputted, but we also detected a limited but detectable amount of PCR slippage. The use of a synthetically designed template allowed a direct measurement of polymerase slippage artifacts in the serine repeat region. This region is used for classifying *Cryptosporidium* subtypes, depending on the number and variety of serine repeats. We observed slippage in both templates, with a consistently higher rate in the *C parvum* amplicon. Since our template samples had a different number of repeats (13 in *C hominis* and 20 in *C parvum*), this corroborates observations in previous studies where slippage frequency is directly increased with the length of the serine repeat region [10]. Our study used Platinum Taq DNA polymerase, which has 1X fidelity versus Taq; however, future studies could test results using higher-fidelity enzymes. Nonetheless, estimations of the expected rates of error in the serine repeat region can be extrapolated from our data, are in agreement with previously observed patterns for slippage rates in trinucleotide repeat regions [11], and may be further refined with future research using different-sized repeat regions relevant to different *Cryptosporidium* species and subtypes.

As anticipated, the post-PCR combinations of *C hominis* and *C parvum* PCR products closely matched the expected ratios. However, we detected a marked bias toward rare taxa in the pre-PCR communities. For example, in the 99:1 scenario, the observed number of reads was approximately 70:30. A bias toward rare or low copy number taxa has been observed previously in nested PCR studies when more cycles were applied in the first round of PCR on relatively highly diverse communities [12]. However, in more complex assemblages these patterns are less predictable [13, 14] and may also vary in real samples with much lower amounts of *Cryptosporidium* DNA template as well as PCR inhibitors. Amplicon slippage rates did not differ under any of the different treatments, including those with lower initial template concentrations.

Our results were similar using dada2 and sequence analyses by Geneious, but it is important to note that under the default dada2 settings, many of the variants present in the samples were forced to cluster together around the most common fragment length(s). The degree of clustering was dependent on the variation in the community, with lower diversity—that is, 1 subtype more prone to clustering effects. The appropriate BANDSIZE settings must be used, based on expected error rates given the size of the repeat region. This will help past and future studies separate real, biological variation inside the host from artifacts of the PCR and sequencing process.

It is possible that the replication slippage rates observed in the study are not attributable to PCR error alone. Since the synthetically generated templates used in the study were inserted into the pUC57 plasmid, slippage DNA replication as part of normal cloning might have contributed to the observed results as well as during PCR. However, replication slippage during cloning is expected to be low and previous observations using *Cryptosporidium* templates suggest low error rates in templates with clonal plasmid vectors [10].

While our analyses were conducted on synthetic DNA, it is important to note that slippage is a natural phenomenon. This process holds the potential to foster repeat variations in specific sites, thereby aiding *Cryptosporidium* in evading immune responses triggered by prior infections. Despite recent research demonstrating that the repeat regions in *Cryptosporidium* gp60, on which our study is based, do not appear to impact antibody recognition [15], our research has implications for studies assessing the diversity of PCR-amplified repeat regions.

Our study shows that PCR slippage during amplification leads to significant error rates in metabarcoding sequencing of amplicons with repeat regions. The rate of error appears proportional with size of the repeat region. In addition, our study shows a tendency for overrepresentation of rare taxa in mixed assemblages. Contamination from different subtype families was absent. Taken together, our results indicate that many subtypes seen in metabarcoding studies are likely artifacts resulting from PCR slippage during amplification and may be distributed in predictable ways, enabling better interpretation of results. Mixtures of different subtype families and species in *Cryptosporidium* metabarcoding studies do likely therefore represent real within-sample variation.

## Supplementary Data

Supplementary materials are available at *The Journal of Infectious Diseases* online (http://jid.oxfordjournals.org/). Supplementary materials consist of data provided by the author that are published to benefit the reader. The posted materials are not copyedited. The contents of all supplementary data are the sole responsibility of the authors. Questions or messages regarding errors should be addressed to the author.

## Supplementary Material

jiae065_Supplementary_Data

## Notes

***Financial support***. This work was supported by Royal Society Te Apārangi (grant number RDF-MAU170 to D. T. S. H.); the New Zealand Ministry of Health (contract number 355766-02 to D. T. S. H.); and the Percival Carmine Chair in Epidemiology and Public Health, Massey University (to D. T. S. H.).

## References

[1] Viguera E , Canceill D, Ehrlich SD. Replication slippage involves DNA polymerase pausing and dissociation. EMBO J 2001; 20:2587–95. 11350948 10.1093/emboj/20.10.2587 PMC125466

[2] Balzano E , Pelliccia F, Giunta S. Genome (in)stability at tandem repeats. Semin Cell Dev Biol 2021; 113:97–112. 33109442 10.1016/j.semcdb.2020.10.003

[3] Xiao L , Feng Y. Molecular epidemiologic tools for waterborne pathogens *Cryptosporidium* spp. and *Giardia duodenalis*. Food Waterborne Parasitol 2017; 8–9:14–32. 10.1016/j.fawpar.2017.09.002 PMC7034008 32095639

[4] Braima K , Zahedi A, Egan S, et al Molecular analysis of cryptosporidiosis cases in Western Australia in 2019 and 2020 supports the occurrence of two swimming pool associated outbreaks and reveals the emergence of a rare *C. hominis* IbA12G3 subtype. Infect Genet Evol 2021; 92:104859. 33848684 10.1016/j.meegid.2021.104859

[5] Zahedi A , Gofton AW, Jian F, et al Next generation sequencing uncovers within-host differences in the genetic diversity of *Cryptosporidium* gp60 subtypes. Int J Parasitol 2017; 47:601–7. 28495122 10.1016/j.ijpara.2017.03.003

[6] Bailly E , Valot S, Vincent A, et al Evaluation of next-generation sequencing applied to *Cryptosporidium parvum* and *Cryptosporidium hominis* epidemiological study. Pathogens 2022; 11:938. 36015058 10.3390/pathogens11080938 PMC9414878

[7] Shinde D , Lai Y, Sun F, Arnheim N. Taq DNA polymerase slippage mutation rates measured by PCR and quasi-likelihood analysis: (CA/GT)n and (A/T)n microsatellites. Nucleic Acids Res 2003; 31:974–80. 12560493 10.1093/nar/gkg178 PMC149199

[8] Illumina Inc. Preparing 16S ribosomal RNA gene amplicons for the Illumina MiSeq System. 16S Metagenomic Sequencing Library Preparation Manual. San Diego, CA: Illumina Inc, 2013.

[9] Callahan BJ , McMurdie PJ, Rosen MJ, Han AW, Johnson AJ, Holmes SP. DADA2: high-resolution sample inference from Illumina amplicon data. Nat Methods 2016; 13:581–3. 27214047 10.1038/nmeth.3869 PMC4927377

[10] Dettwiler I , Troell K, Robinson G, et al TIDE analysis of *Cryptosporidium* infections by gp60 typing reveals obscured mixed infections. J Infect Dis 2022; 225:686–95. 34417806 10.1093/infdis/jiab417

[11] Lai Y , Sun F. The relationship between microsatellite slippage mutation rate and the number of repeat units. Mol Biol Evol 2003; 20:2123–31. 12949124 10.1093/molbev/msg228

[12] Yu G , Fadrosh D, Goedert JJ, Ravel J, Goldstein AM. Nested PCR biases in interpreting microbial community structure in 16S rRNA gene sequence datasets. PLoS One 2015; 10:e0132253. 26196512 10.1371/journal.pone.0132253 PMC4509648

[13] Bohmann K , Elbrecht V, Carøe C, et al Strategies for sample labelling and library preparation in DNA metabarcoding studies. Mol Ecol Resour 2022; 22:1231–46. 34551203 10.1111/1755-0998.13512 PMC9293284

[14] Kelly RP , Shelton AO, Gallego R. Understanding PCR processes to draw meaningful conclusions from environmental DNA studies. Sci Rep 2019; 9:1–14. 31431641 10.1038/s41598-019-48546-x PMC6702206

[15] Gilchrist CA , Campo JJ, Pablo JV, et al Specific *Cryptosporidium* antigens associate with reinfection immunity and protection from cryptosporidiosis. J Clin Invest 2023; 133: e166814. 37347553 10.1172/JCI166814 PMC10425216

