# Supplementary material for: Quantifying Replication Slippage Error in Cryptosporidium Metabarcoding Studies
Source: J Infect Dis. 2024 Feb 8;230(1):e144–8. doi: 10.1093/infdis/jiae065 (PMC11272095; doi:10.1093/infdis/jiae065)
Supplement: jiae065_Supplementary_Data [file jiae065_supplementary_data.zip › Supplemental Figure Legend.docx]

Supplementary Figure 1. Replication slippage rates for *C. hominis* and *C. parvum* as indicated by the distribution of reads with the expected number (0), missing (-1) or gaining (1) a trinucleotide repeat.
