# Supplementary figures and images for: Quantifying Replication Slippage Error in Cryptosporidium Metabarcoding Studies
Source: J Infect Dis. 2024 Feb 8;230(1):e144–8. doi: 10.1093/infdis/jiae065 (PMC11272095; doi:10.1093/infdis/jiae065)

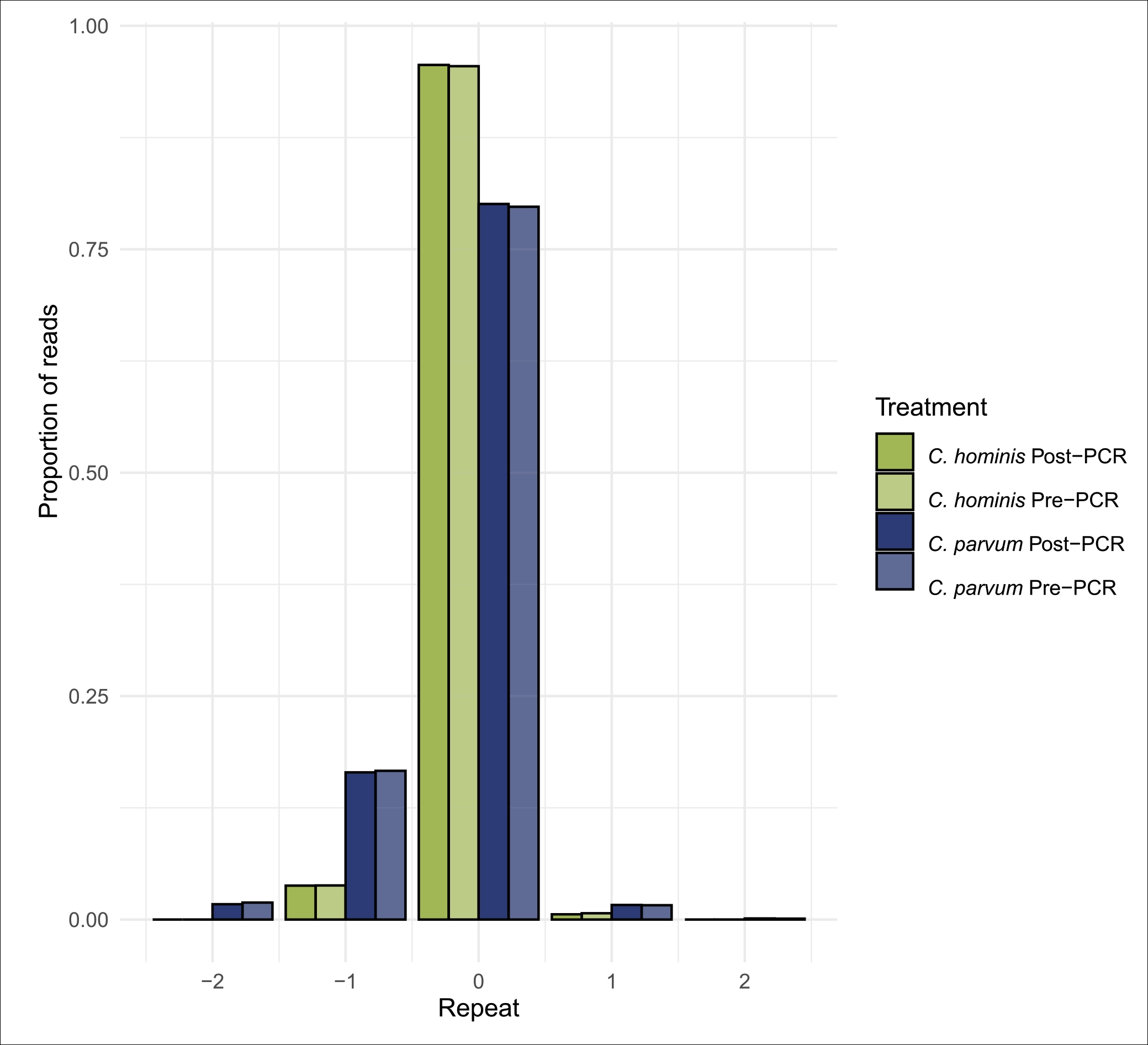

Supplement: jiae065_Supplementary_Data [file jiae065_supplementary_data.zip › SI_fig1.jpg]
